# Trametinib as a targeted treatment in cardiac and lymphatic presentations of Noonan syndrome

**DOI:** 10.3389/fped.2025.1475143

**Published:** 2025-02-18

**Authors:** Isabel De Brouchoven, Juan Lorand, Léon Bofferding, Arthur Sorlin, An Van Damme, Olivier Danhaive

**Affiliations:** ^1^ Division of Neonatology, Saint-Luc University Hospital, UCLouvain, Brussels, Belgium; ^2^ Division of Neonatology, Kannerklinik, Luxembourg Hospital Center, Luxembourg, Luxembourg; ^3^ National Center of Genetics (NCG), Laboratoire National de Santé (LNS), Dudelange, Luxembourg; ^4^ Division of Pediatric Haemato-Oncology and Centre for Vascular Anomalies, Saint-Luc University Hospital, UCLouvain, Brussels, Belgium; ^5^ Department of Pediatrics, University of California San Francisco, San Francisco, CA, United States

**Keywords:** Noonan syndrome, RASopathy, MEK inhibitors, trametinib, congenital pulmonary lymphangiectasia, chylothorax, hypertrophic cardiomyopathy, pulmonary valve stenosis

## Abstract

**Introduction:**

Rare pathogenic variants in the *PTPN11, KRAS, SOS1* and *RAF1* genes are the main molecular causes of Noonan syndrome (NS). Most are dominant gain-of-function variants that cause an overactivation of the RAS/MAPK signaling pathway leading to uncontrolled cell proliferation in many organs and systems. Albeit phenotypically heterogeneous, NS can be associated with severe cardiovascular and lymphatic anomalies, potentially lethal during infancy, neonatal and fetal periods. MEK inhibitors, a class of drugs targeting the final steps of the RAS/MAPK pathway and originally developed for cancer therapy, have been tested in preclinical studies as a targeted treatment for NS. These studies led to the occasional off-label use of MEK inhibitors in patients with RASopathies.

**Methods:**

We report the case of a preterm infant with congenital pulmonary lymphangiectasis, chylothorax and hypoxic respiratory failure refractory to conventional management, who was treated with trametinib after identification of a NS *PTPN11* class 5 variant. We performed a systematic review of the current published evidence on trametinib efficacy and safety for severe respiratory and/or cardiac manifestations in infants and children with Noonan syndrome, querying PubMed, Embase, Cochrane and Scopus databases, following the PRISMA guideline for systematic reviews, and using the Joanna Briggs Institute (JBI) Critical Appraisal tool for quality assessment of published evidence.

**Results:**

In our patient, a five-week trametinib course, maximum dose 0.025 mg/kg/day, led to chylothorax resolution and gradual pulmonary function improvement, allowing extubation to non-invasive support, discharge home at a corrected age of 4 months, and weaning off home oxygen therapy by 10 months. No formal clinical trial of trametinib in neonatal/pediatric Noonan syndrome has been published to our knowledge. We collected 16 published cases, and added this case for reviewing trametinib regimen, efficacy and safety. A short-term improvement of symptoms was reported in all cases, with three deaths presumably unrelated to trametinib. Moderate side effects were reported in a subset of patients. Long-term follow-up data were not available.

**Discussion:**

Trametinib is a promising drug in NS. Clinical trials are warranted to establish safety, efficacy, and standardized protocols for the use of trametinib as a rescue therapy in critically ill children and explore its potential place in the treatment of various NS comorbidities.

**Systematic Review Registration:**

clinicaltrials.gov, identifier [NCT06555237].

## Introduction

Noonan syndrome (NS) is a common autosomal dominant genetic disorder with multisystemic manifestations, including facial dysmorphism, short stature, congenital heart disease (CHD), renal anomalies, orthopedic defects, neurological, neurosensorial and developmental issues, gastrointestinal, hematologic and endocrine dysregulations, and lymphatic disorders among others (1). NS occurs in approximately 1 in 1,000 to 2,500 live births and is the most prevalent syndrome among RASopathies, a group of rare diseases characterized by upregulation of the ubiquitous RAS/MAPK signaling pathway, resulting in complex developmental disorders and neoplasia (2–6).

The phenotype varies from oligosymptomatic adults without significant medical issues to severely affected neonates with life-threatening heart disease and lymphatic defects.

CHD has a prevalence as high as 80% in NS, the most frequent forms being pulmonary stenosis (PS), atrial septal defects and other valve dysplasias (mitral valve, aortic vavle). It is common for these defects to occurring together, but can be isolated, and complex CHD such as balanced and unbalanced AVSD can also be seen. Additionally, hypertrophic cardiomyopathy (HCM) is frequently present in specific genotypes and may coexist with these heart conditions (7–9). Lymphatic anomalies are among the most common fetal features of RASopathies, observed in up to 20% NS subjects at birth, and up to 37% over the lifespan (4, 10–12). A spectrum of complex anomalies such as enlargement of lymphatic channels (lymphangiectasia), dysmotility, or central conducting lymphatic anomaly (CCLA) cause inadequate clearing of lymph with resultant stasis and reflux, affecting various organ systems, and resulting in lymphedema, chylothorax, pericardial effusion, congenital pulmonary lymphangiectasia (CPL), exudative enteropathy, chylous ascites, cystic hygroma, hydrops and other manifestations, some of which are detectable as early as the second trimester of pregnancy and are potentially life-threatening in neonates (12–14).

**Figure 1:**
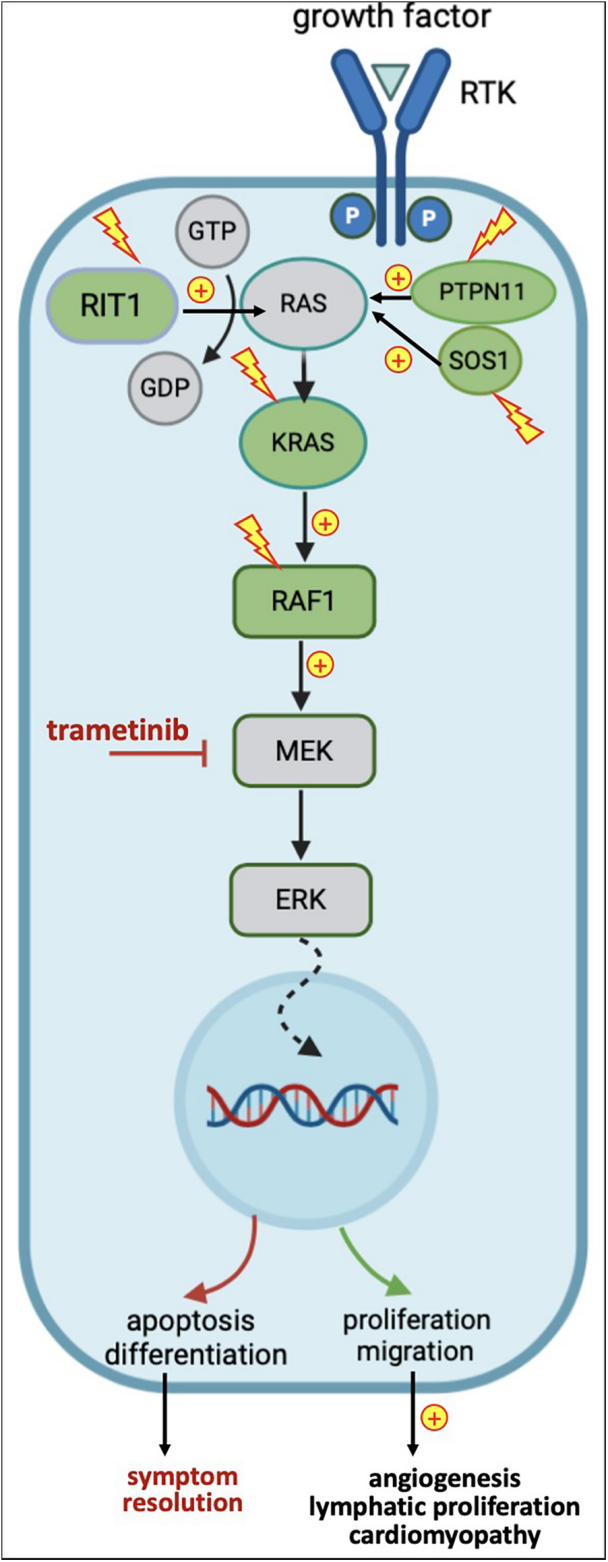
Simplified representation of the RAS-MAPK pathway, genes affected by variants in noonan syndrome, and downstream effects of variants and trametinib. In green: genes upregulated in NS; in yellow: activating variants; in red: inhibitory effects.

NS is heterogeneous both phenotypically and genetically. Over 50% of NS patients carry gain-of-function variants in the *PTPN11* gene. Less frequently, pathogenic variants can be found in SOS1, *RIT1, RAF1* and *KRAS* genes, and even more rarely in *BRAF, LZTR1, MAP2K1, NRAS*, and others (6, 10, 15, 16). These variants affecting mediators and regulators of the RAS/MAPK cascade result in uncontrolled up-regulation of several target genes implicated in cell survival, metabolism, proliferation, and differentiation, responsible of the various clinical manifestations (Figure 1) (5). *PTPN11* and *SOS1* variants are less often associated with HCM, in contrast to *RAF1* and *RIT1* variants*. RAF1* variants are associated with severe cardiac outcome, especially in the neonatal period (15, 17). *SOS2* and *RIT1* variants show a higher prevalence of lymphatic anomalies (11, 12, 18). There are overlaps between the phenotypes and genotypes of NS and other RASopathies (7). Next-generation sequencing (NGS) panels and whole-exome sequencing (WES) have a yield up to 90%, leaving only a small subset of clinically diagnosed NS patients without a definite molecular diagnosis (19).

The mainstay of NS management in neonates and infants relies on treating critical hemodynamic and respiratory failures with respiratory and hemodynamic support. Symptomatic treatments for HCM and CHD include diuretics, beta-blockers and surgery. CPL, CCLA and chylothorax can benefit from combinations of parenteral nutrition, low-fat, medium-chain triglyceride-based diet, somatostatin and analogues, chest drains, thoracic duct surgery and others (1, 20, 21). However, over the past two decades, an improved understanding of the pathophysiology underlying RASopathies and specifically NS has fostered multiple research efforts aimed at developing novel agents that restore normal MAPK signaling. As most variants result in an uncontrolled increase of the mitogen signal, compounds that inhibit the final mediators of this cascade have emerged as possible candidates for a precision medicine approach.

MEK inhibitors have initially been developed as cancer therapy, since approximately 20% of malignancies harbor somatic variants causing an overactivation of the RAS/MAPK pathway, making them the second most frequent alteration in cancer (22). As a result, since 2005, several molecules have been approved by the Federal Drug Administration (FDA) for specific oncologic indications (23). *in vitro* and animal models testing the efficacy of MEK inhibitors showed promising results in improving various aspects of RASopathies (15, 24–26). Studies in lymphatic organoids, zebrafish, drosophila and mice have defined molecular pathobiology and identified MEK, a protein kinase acting downstream in the RAS/MAPK signaling pathway (Figure 1), as a therapeutic target in NS (27). These studies demonstrate that lymphatic vessels develop early in embryogenesis, originating from endothelial cells of the cardinal vein that acquire lymphatic identity through SOX18 activation. SOX18, in turn, is directly regulated by ERK, another downstream protein kinase in the RAS/MEK/MAPK signaling pathway. In preclinical mouse studies, gain-of-function variants in RAS/MAPK-associated genes resulted in SOX18 overexpression, leading to excessive lymphatic cell proliferation and congenital lymphangiectasia mimicking NS phenotype. Pharmacological MEK inhibition during embryogenesis suppressed this overactivation of the RAS/MAPK cascade, preventing lymphatic abnormalities and rescuing the NS-like phenotype in animals carrying the pathogenic variants (28). Similarly, other investigators reproduced an NS-like HCM phenotype in RAS/MAPK mutant animals, which could be rescued by fetal MEK inhibition (Figure 1) (15).

These studies have led to off-label clinical use of trametinib, one of the most studied selective allosteric MEK inhibitors, as a compassionate treatment of certain severe or life-threatening manifestations. In children, the use of trametinib for severe NS complications has been only reported in a few single case reports and small series (17, 29–40). We describe the clinical course of a preterm neonate with severe hypoxic respiratory failure due to CPL and chylothorax refractory to conventional therapies, who was successfully and durably treated with a 32-day course of trametinib. In addition, we present a review of clinical reports and series of NS infants and children with severe cardiac and lymphatic manifestations treated with trametinib, with the purpose of analyze the available evidence regarding dosage, regimen, efficacy, safety and adverse events.

## Methods

Clinical data for the case reported here were extracted from electronic medical records. The parents provided informed consent for off-label use of trametinib and the use of anonymized clinical data and imaging for publication.

For the literature review, we collected published clinical reports of infants and children with the following inclusion criteria: age <18 years, genetically confirmed NS diagnosis, trametinib as treatment for severe/critical lymphatic or cardiac complication. We screened the Cochrane Library (RRID:SCR_013000), PubMed (RRID:SCR_004846), EMBASE (RRID:SCR_001650), Scopus (RRID:SCR_022559) and Google Scholar (RRID:SCR_008878) databases using the following search framework: (Trametinib) AND (Noonan Syndrome) AND (Chylous effusion OR Cardiac diseases OR lymphatic diseases). The literature search was last updated on 27 November 2024. We excluded duplicates (same citation in different databases, or same patient reported in different references, i.e., conference abstract plus full paper), articles not meeting selection criteria and articles that do not provide information about trametinib regiment. We applied the Joanna Briggs Institute (JBI) Critical Appraisal tool (https://jbi.global/sites/default/files/2020-08/Checklist_for_Case_Reports.pdf) in order to assess the quality of the selected reports (Table 1). We retained eleven full articles and two conference abstracts, reporting on sixteen original cases (Appendix 1).

**Table 1:** Quality assessment of case reports.

	De Brouchoven 2024	Toro et al. 2024 (35)	Meisner et al. 2021 (36)	Nakano et al. 2022 (31)	Hribernik et al. 2023 (30)	Andelfinger et al. 2019 (37)	Lioncino et al. 2022 (32)	Leegaard et al. 2022 (38)	Mussa et al. 2021 (17)	Dori et al. 2020 (29)	Taliercio 2024 et al. (Abstract) (34)	Hofbeck et al. 2021 (Abstract) (33)	Pascarella et al. 2024 (39)	Leenders et al. 2024 (40)
Were patient's demographic characteristics clearly described?	Yes	Yes	Yes	Yes	Unclear	Unclear	Yes	Yes	Yes	Yes	Yes	Unclear	Yes	Yes
Was the patient's history clearly described and presented as a timeline?	Yes	Yes	Unclear	No	Yes	Unclear	Yes	Yes	Yes	Yes	Yes	Unclear	Yes	Yes
Was the current clinical condition of the patient on presentation clearly described?	Yes	Yes	Yes	Unclear	Yes	No	Yes	Yes	Yes	Yes	Unclear	Unclear	Yes	Yes
Were diagnostic tests or assessment methods and results clearly described?	Yes	Yes	Yes	Yes	Yes	No	Yes	Yes	Yes	Yes	Unclear	Unclear	Yes	Yes
Was the intervention(s) or treatment procedure(s) clearly described?	Yes	Yes	Yes	Yes	Yes	Yes	Yes	Yes	Yes	Yes	Unclear	Unclear	Yes	Yes
Was the post-intervention clinical condition clearly described?	Yes	Yes	Unclear	Yes	Yes	Yes	Yes	Yes	Yes	Yes	Yes	Yes	Yes	Yes
Were the adverse events (harms) or unanticipated events identified and described?	Yes	Yes	Yes	Unclear	Yes	No	Yes	Yes	Yes	Yes	No	Unclear	Yes	Yes
Does the case report provide takeaway lessons?	Yes	Yes	Yes	Yes	Yes	Yes	Yes	Yes	Yes	Yes	Yes	Yes	Yes	Yes
Score:	16/16	16/16	14/16	12/16	15/16	8/16	16/16	16/16	16/16	16/16	11/16	10/16	16/16	16/16

Johanna Brings Institute (JBI) Critical appraisal checklist for case reports:

Scoring system: YES = 2 points, UNCLEAR = 1 point, NO: 0 point. Green: good quality (16/16 points); yellow: moderate quality (12–15/16 points); red: low quality (<11 points or >2 NO or >3 UNCLEAR).

## Case report

**Figure 2:**
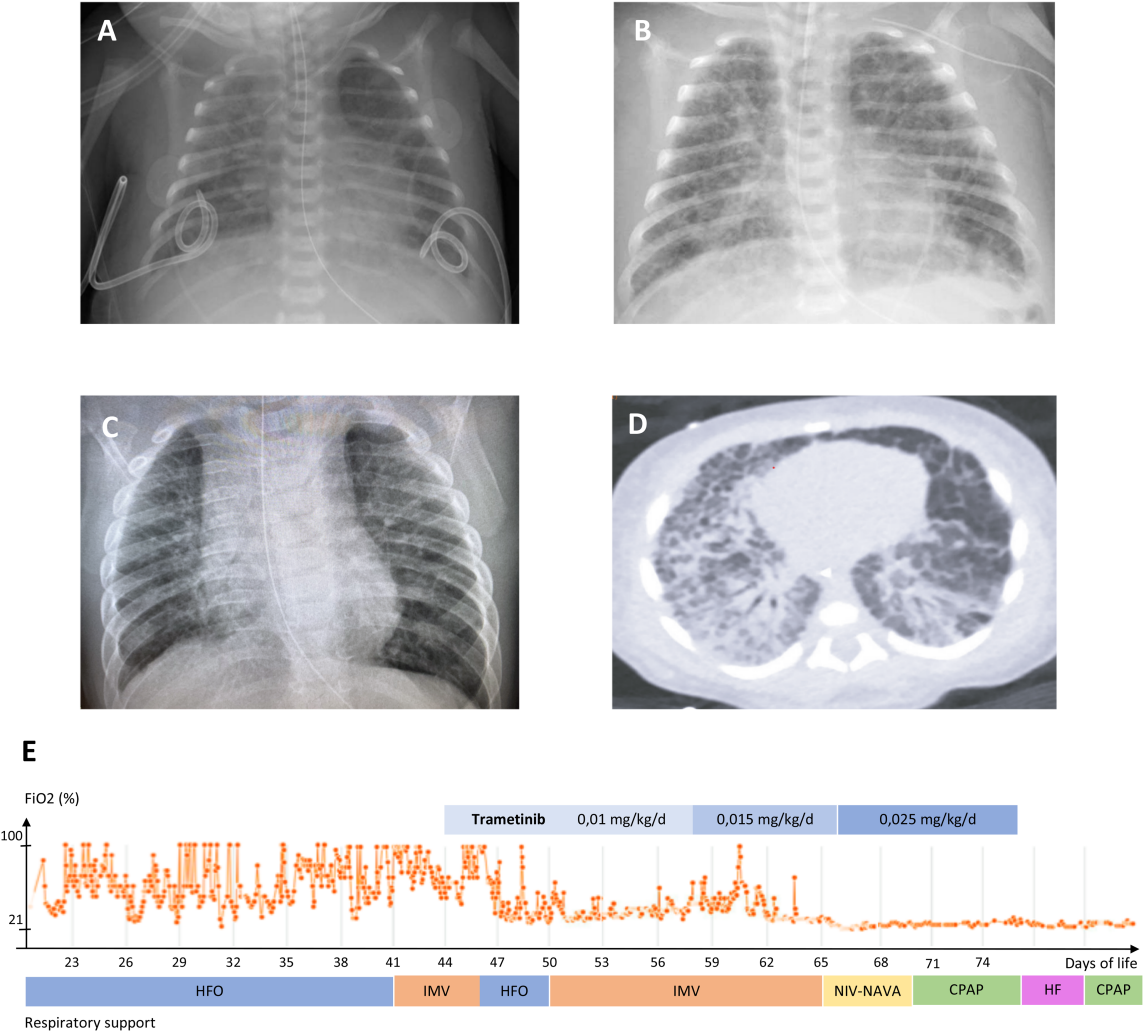
**(A**–**C)** chest radiograms of patient #1: **(A)** 22 days of life; **(B)** 2 months; **(C)** 12 months. **(D)** Chest computed tomography scan, 32 days of life. **E**: mean daily FiO_2_ during level 4 neonatal intensive care stay, ventilation mode and trametinib regimen. HFO: high frequency oscillatory ventilation; IMV: intermittent mandatory ventilation; NIV-NAVA: non-invasive neurally-adjusted ventilatory assist; CPAP: continuous positive airway pressure; HF: high-flow nasal cannula.

The infant was a 32-week preterm male born to a 31-year-old G2P1>2 woman with an unremarkable medical and family history, delivered by emergency cesarean section for acute fetal distress. Pre-delivery fetal ultrasonography revealed massive hydrops and bilateral pleural effusions. The Apgar score was 0, 3 and 5 at 1, 5 and 10 min of life. Bilateral chest tubes were placed during resuscitation and the infant was transferred to a level III neonatal intensive care unit. Birth weight was 2.8 Kg (z-score 2.46). The infant presented with hypotension, hypoxic respiratory failure, dysmorphic features including broad forehead, low-set ears, hypertelorism, down-slanted narrow palpebral fissures and a flat nasal bridge. The subsequent workup demonstrated hypertrophic cardiomyopathy and chylothorax. The infant received continuous invasive respiratory support and vasopressors for 5 days, and was treated with octreotide and propranolol from the second week of life, with no significant improvement. He was transferred to our level IV NICU on day-of-life 21, still on high ventilatory setting and with continuous pleural drainage (Figure 2).

A chest CT was performed, showing typical features of congenital pulmonary lymphangiectasis combined with ventilator-induced lung injury. Repeat echocardiography showed a marked concentric bi-ventricular myocardial hypertrophy with no outflow obstruction, and mild, infra-systemic pulmonary hypertension. Thus, the infant was treated with somatostatin from day 22 to 34, which led to decrease thoracic chylous output and chest tubes removal by day 28. However, severe respiratory failure persisted, with oxygenation indices ranging between 16.5 and 30.2, despite a course of dexamethasone (4 mg/kg cumulative dose) from day 28 to 37, requiring high-frequency ventilation and inhaled nitric oxide (up to 20 ppm). The infant suffered several comorbidities including hypoalbuminemia, fluid retention, nephrocalcinosis, transient cholestasis, deep vein thrombosis, and required heavy prolonged sedation with combinations of fentanyl, methadone, clonidine, dexmedetomidine and midazolam.

A RASopathy NGS panel undertaken at the referring hospital revealed presence at a heterozygous state of the c.188A>G (p.Tyr63Cys) class 5 variant of the *PTPN11* gene (rs121918459), located in a hotspot and found in several subjects with Noonan syndrome (41). The parents did not carry the variant. Upon reception of the genetic result on day 34, given the severity of respiratory failure and the lack of response to conventional treatments, we sought authorization for off-label use of trametinib to the pharmaceutical company (Novartis Pharma NV/SA, Vilvoorde, Belgium), which was granted on day 45. Trametinib was started PO at 0.01 mg/kg/day q24 for 14 days, then increased to 0.015 mg/kg/day for 8 days, then to 0.025 mg/kg/day for 10 days. As a response, oxygen and ventilatory support could rapidly be weaned, iNO was discontinued and the infant was extubated on day 62 to non-invasive neurally-adjusted ventilatory assist (NAVA), then CPAP. After the onset of trametinib, we observed some emesis, which responded to ondansetron. On day 75, the baby presented with trametinib-related colitis. The medication was discontinued, and the infant was transferred to the hospital of origin. A gastrostomy with Nissen fundoplicature was performed for persisting oral motor dysfunction and gastroesophageal reflux. He was discharged home on 0.5 L/min oxygen at day 176, corrected age 4 months 1 week. Systematic reporting of drug response and adverse events were submitted to the Managed Access Program (MAP) & Post-Study Drug Supply (PSDS) to the Novartis Patient Safety Department.

At the latest follow-up visit at 10 months corrected age, he was still on oxygen 0.1l /min during nighttime which could subsequently be stopped. Somatic growth, neurodevelopment, hearing and vision are in the normal range. Echocardiography shows only a mild pulmonary valve stenosis with no significant gradient, no hypertrophic cardiomyopathy, and no pulmonary hypertension. He was still presenting major oral motor dysfunction and was mostly fed by gavage.

## Review of published cases

Patients’ phenotype, genotype and clinical course are summarized in Table 2. The genes involved are *SOS1* in five patients, *RIT1* in five, *PTPN11* in five and *RAF1* in two. No correlation appears between the dominant symptoms and specific genes. Whereas dysmorphic features were noticeable since birth, the onset of critical cardiac and/or lymphatic complications is variable, as 13/17 patients presented in the neonatal period and 4/17 (Dori 2020, Hribernik 2023, Nakano 2020 case 1, Taliercio 2024 case 2) later in infancy, between 3 months and 5 years of age. In 8/17 patients (De Brouchoven 2024, Dori 2020, Hirbernik 2023, Nakano 2022 cases 1 and 2, Leenders 2024, Hofbeck 2021, Taliercio 2024), the clinical course was dominated by severe lymphatic manifestations, which triggered the compassionate use of trametinib. Among these, refractory chylothorax presented after thoracic surgery in 3/8 patients (Nakano 2022 cases 1 and 2; Hofbeck 2021). 6/17 patients (Pascarella 2024, Lioncino 2022, Nakano 2022 case 3, Torok 2024, Meisner 2021, Andelfinger 2019 case 2) showed a combination of severe lymphatic and cardiac manifestations. 3/17 patients (Andeflinger 2019 case 1, Leegaard 2022, Mussa 2021) received trametinib primarily for cardiac manifestation unresponsive to conventional treatments.

**Table 2a:** Clinical presentation.

#Case	Sex	GA(w)	Genetic variant			Primary indication for MEK inhibition therapy	
			Gene	cDNA	Protein change	Lymphatic diseases (age of onset)	Cardiac diseases (age of onset)
#1 (De Brouchoven 2024)	M	32	*PTPN11*	c.188A>G	p.(Tyr63Cys)	CPL with Chylothorax (at birth) Lymphoedema	/
#2 (Dori 2020)	F	28	*SOS1*	c.2536G>A	p.(Glu846Lys)	CCLA with Chylothorax (5.2 years) PIL with GI Bleeding (14 years)	/
#3 (Hribernik 2023)	F	N/A	*RIT1*	N/A	N/A	CPL with Chylothorax (2.7 years)	/
#4 (Nakano 2022, case 1)	F	N/A	*RIT1*	c.246T>G	p.(Phe83Leu)	CCLA Post-surgical chylothorax (4 years) Lymphoedema	/
#5 (Nakano 2022, case 2)	F	27	*SOS1*	c.1322G>A	p.(Cys441Tyr)	CCLA CPL Post-surgical chylothorax (3 months) Chylous ascites Lymphoedema	/
#6 (Leenders 2024)	M	31	*PTPN11*	c.922A>G	p.(Asn308Asp)	CCLA with chylothorax (at birth)	/
#7 (Hofbeck 2021)	F	NA	*PTPN11*	c.854T>C	p.(Phe285Ser)	Post-surgical chylothorax (5.4 years) Lymphoedema	/
#8 (Taliercio 2024, case 2)	F	29	*SOS1*	c.2536G>A	p.(Glu846Lys)	Chylothorax (3 months)	/
#9 (Pascarella 2024)	F	37	*PTPN11*	c.854T>C	p.(Phe285Ser)	Chylothorax (at birth)	HCM (at birth) PVS, RVOTO
#10 (Lioncino 2022)	M	34	*SOS1*	c.1655G>C	p.(Arg552Tyr)	CPL with Chylothorax (at birth)	MAT
#11 (Nakano 2022, case 3)	M	N/A	*PTPN11*	c.854T>C	p.(Phe285Ser)	CPL and CCLA with Chylothorax	HCM (4 months) PVS
#12 (Torok 2024)	F	Term	*SOS1*	c.508A>G	p.(Lys170Glu)	Chylothorax (at birth) Chylous ascites	HCM, LVOTO (6 weeks) PVS (6 weeks) ASD PA stenosis MV dysplasia
#13 (Meisner 2021)	F	39	*RAF1*	c.770C>T	p.(Ser257Leu)	Chylothorax (6 weeks)	MAT (3 days) HCM (3 days) PVS
#14 (Andelfinger 2019, case 2)	F	36	*RIT1*	c.246T>G	p.(Phe82Leu)	Chylothorax (3 months)	HCM, RVOTO/LVOTO (at birth) PVS
#15 (Andelfinger 2019, case 1)	N/A	38	*RIT1*	c.140 G>C	p.(Ser35Thr)	/	HCM (3 weeks) PVS, AVS (3 weeks) MV/TV dysplasia
#16 (Leegaard 2022)	F	37	*RIT1*	c.170C>G	p.(Ala35Gly)	/	HCM (at birth) RVOTO PVS MV dysplasia
#17 (Mussa 2021)	F	33	*RAF1*	c.770C>T	p.(Ser257Leu)	/	HCM (at birth) RVOTO/LVOTO PV dysplasia

**Table 2b:** Other clinical manifestations.

#Case	Other complications of Noonan syndrome not directly targeted by MEK inhibition therapy		Comorbidity
	Cardiac diseases	Other diseases	
#1 (De Brouchoven 2024)	HCM PVS		Respiratory failure Pulmonary hypertension Hypogammaglobulinemia
#2 (Dori 2020)		Intestinal vascular ectasia with PLE GH deficiency Failure to thrive	VSD
#3 (Hirbernik 2023)	HCM, RVOTO/PVS LVOTO		Distal ileum perforation Hypogammaglobulinemia Multiple sepsis ASD
#4 (Nakano 2022, case 1)	HCM PVS MV dysplasia		Esophageal atresia Respiratory failure Pulmonary hypertension Hypogammaglobulinemia
#5 (Nakano 2022, case 2)	PVS	Failure to thrive	Respiratory failure Immunodeficiency Coagulopathy
#6 (Leenders 2024)			Polyhydramnios Respiratory failure Hypogammaglobulinemia Sepsis Hypothyroidism
#7 (Hofbeck 2021)	PVS AVS HCM, RVOTO		Respiratory failure
#8 (Taliercio 2024, case 2)	PVS HCM, RVOTO		Polyhydramnios Respiratory failure
#9 (Pascarella 2024)			Polyhydramnios HIE
#10 (Lioncino 2022)	HCM PVS		Polyhydramnios Respiratory failure
#11 (Nakano 2022, case 3)		Myeloproliferative disorder	Respiratory failure
#12 (Torok, 2024)			Polyhydramnios Pulmonary hypertension
#13 (Meisner 2021)			Respiratory failure Two cardiac arrests Bronchomalacia
#14 (Andelfinger 2019, case 2)			Polyhydramnios Pulmonary hypertension
#15 (Andelfinger 2019, case 1)			Polyhydramnios
#16 (Leegaard 2022)			Polyhydramnios
#17 (Mussa 2021)			IVH Post-hemorrhagic hydrocephalus Pulmonary Hemangiomatosis Polyhydramnios

ASD, atrial septal defect; AVS, aortic valve stenosis; CCLA, central conducting lymphatic anomaly; CPL, congenital pulmonary lymphangiectasis; GA, gestational age; GI, gastrointestinal; GH, growth hormone; HCM, hypertrophic cardiomyopathy; HIE, hypoxic-ischemic encephalopathy; IVH, intraventricular hemorrhage; LVOTO, left ventricle outflow tract obstruction; MAT, multifocal atrial tachycardia; MV, mitral valve; PLE, protein-losing enteropathy; PVS, pulmonary valve stenosis; RVOTO, right ventricle outflow tract obstruction; VSD, ventricular septal defect; N/A: not available; PA: pulmonary artery; PIL, primary intestinal lymphangiectasia; PV, pulmonary valve; RVOTO, right ventricle outflow tract obstruction; TV, tricuspid valve.

Trametinib therapy is summarized in Table 3. The median age at the start of the treatment was 3.5 months (0.75 m–84 m) in the neonatal-onset group and 22 months (3 m–178 m) in the infant group. The median time between the onset of the critical symptoms and the start of treatment was 4 months (0.75 m–106.8 m). Trametinib was administered orally once a day with an initial dose ranging from 0.01 to 0.032 mg/kg/day. In 3/17 patients (De Brouchoven 2024; Dori 2020; Hofbeck 2021) the dosage was gradually increased based on clinical response and tolerance, to a maximum of 0.03 mg/kg/day. In 4/17 patients (Nakano2022, case 1–3; Leenders 2024) trametinib treatment was scaled up in frequency (i.e., initially q48 h, then q36, then q24) rather than in dosage. The duration of the treatment varied between reports, with a median of 9 months (0.6 m–24 m).

**Table 3:** Conventional and trametinib treatments.

#Case	Symptomatic treatments	Procedures	Onset, indication and trametinib course	Clinical response	Side effects	Outcome
#1 (De Brouchoven 2024)	TPN, NPO Low fat diet Octreotide Steroids Respiratory support IVIg iNO	Chest tube	5 weeks CPL 0.010 mg/kg/day × 2 weeks 0.015 mg/kg/day × 8 days 0.025 mg/kg/day × 2 weeks Stop for side effects	Chylothorax resolved CPL improved PVS improved	GI bleeding	10 months follow-up, post-trametinib: home oxygen therapy
#2 (Dori 2020)	Low-fat diet Octreotide PRBC Albumin Steroids	Central lymphatic embolization Duodenal mucosal cauterization	14 years Intestinal lymphangiectasia 0.01 mg/kg/day × 1 weeks 0.02 mg/kg/day × 6 months	CCLA improved Chylothorax resolved GI bleeding resolved	No side effects	6-months follow-up on trametinib: stable at home, improved growth
#3 (Hirbernik 2023)	TPN, NPO–Low-fat diet Octreotide IVIg	RVOT/LVOT myectomy (22 m) Chest tube (2.7 years) Ileostomy	3.33 years Chylothorax 0.032 mg/kg/day × 3 months Therapy completed	Chylothorax resolved Regular diet	Diffuse eczema	8-months follow-up, post trametinib: stable at home
#4 (Nakano 2022, case 1)	Low-fat diet Octreotide Sirolimus Respiratory support	LVOT myectomy MV prothesis Chest tube	4 years Post-surgical chylothorax 0.018 mg/kg/q48h × 3 days 0.018 mg/kg/q36h × 3 days 0.018 mg/kg/day × 2 years	HCM resolved by surgery Chylothorax resolved Regular diet	No side effects	1-year follow-up, on trametinib: Stable at home, improved growth
#5 (Nakano 2022, case 2)	Low-fat diet Octreotide Respiratory support	Esophageal reanastomisis Chest tube	3 months Post-surgical chylothorax 0.025 mg/kg/q48h × 3 days 0.025 mg/kg/q36h × 3 days 0.025 mg/kg/day × 6 months 0.0125 mg/kg/day × 6 Therapy completed	CCLA and chylothorax resolved Regular diet PVS improved	Diffuse eczema	1-years follow-up, end of trametinib course: Stable at home, improved growth
#6 (Leenders 2024)	TPN, NPO Low-fat diet Octreotide Propranolol IVIg Respiratory support	Chest tube	3 weeks Chylothorax 0.018 mg/kg/q48h × 15 days 0.018 mg/kg/day × 12 months Therapy completed	CCLA and chylothorax resolved Regular diet	Diffuse eczema	6-months follow-up, post-trametinib: Stable at home
#7 (Hofbeck 2021)	Respiratory support Diuretics	Pleurodesis RVOT myectomy AV/PV prothesis (5.4 years) Tracheostomy	7 years Post-surgical chylothorax 0.01 mg/kg/day × 7 days 0.02 mg/kg/day × 9 months	Chylothorax resolved Lymphoedema improved LVOTO/RVOTO resolved by surgery	Not reported	9-months follow-up on trametinib: stable at home, NYHA II
#8 (Taliercio 2024, case 2)	Respiratory support	Balloon valvuloplasty RVOT myectomy PV repair Chest tube (3 month)	3 months Chylothorax 0.025 mg/kg/day × 17 days	Chylothorax persistence Chylous ascites HCM, RVOTO resolved by surgery	Not reported	On day 17 of treatment: Redirection of care, death.
#9 (Pascarella 2024)	TPN, NPO Octreotide Respiratory support Therapeutic hypothermia	Chest tube Balloon valvuloplasty	4 months Chylothorax 0.025 mg/kg/day × 18 months	Chylothorax resolved HCM improved PVS persisted	Diffuse eczema	18-months follow-up, on trametinib: Stable at home
#10 (Lioncino 2022)	Propranolol Amiodarone Flecainide Steroids Surfactant Respiratory support	Chest tube	9 weeks Chylothorax and MAT 0.02 mg/kg/day × 4 months	Chylothorax and CPL resolved MAT resolved	No side effects	4-months follow up, on trametinib: Stable at home, G-tube
#11 (Nakano 2022, case 3)	Respiratory support		4 months HCM, Chylothorax 0.023 mg/kg/q48h × 3 days 0.023 mg/kg/q36h × 3 days 0.023 mg/kg/day × 1 months	CPL and chylothorax improved Off respiratory support HCM improved Myeloproliferative disorder resolved	No side effect	“Several weeks” post trametinib: sudden death at home
#12 (Torok 2024)	NPO, TP Octreotide	Chest tube Peritoneal tube PA stent	4 months LVOT, chylous ascites, chylothorax 0.02 mg/kg/day × 14 months	Chylothorax and ascites resolved HCM, MV dysplasia, PA stenosis improved Regular diet	No side effect	14-months follow-up, on trametinib: stable at home
#13 (Meisner 2021)	β-blockers, Amiodarone, Diltiazem, Flecainide Home oxygen	Chest tube	20 weeks Refractory MAT and progressive HCM 0.025 mg/kg/day Stop 3 days for side effects 0.01875 mg/kg/day	Chylothorax resolved MAT improved HCM improved	Diarrhea	9-months follow-up on trametinib: stable
#14 (Andelfinger 2019, case 2)	Propranolol Respiratory support	Chest tube Balloon PA valvuloplasty	13 weeks HCM, chylothorax 0.027 mg/kg/day × 17 months	RVOTO/LVOTO improved Chylothorax resolved	Not reported	17-months follow-up on trametinib: stable at home Improved growth
#15 (Andelfinger 2019, case 1)	Propranolol		14 weeks HCM 0.02 mg/kg/day × 17 months	HCM improved	Not reported	17-months follow-up on trametinib: stable at home–Improved growth
#16 (Leegaard 2022)	Respiratory support	Balloon PV dilatation	6 months HCM 0.025 mg/kg/day × 2 years	HCM resolved	Diffuse Eczema	2-years follow-up on trametinib: stable, home Improved growth
#17 (Mussa 2021)	β-blockers Respiratory support	Ventriculo-peritoneal shunt	6 weeks HCM 0.022 mg/kg/day × 1 months	Transient cardiac function improvement, relapse after neurological surgery	Not reported	On day 46 of treatment: Death Pulmonary hemangiomatosis PAH

AV, aortic valve; CCLA, central conducting lymphatic anomaly; CPL, congenital pulmonary lymphangiectasis; GI, gastrointestinal; HCM, hypertrophic cardiomyopathy; iNO, inhaled nitric oxide; IVIg, intravenous immunoglobulin; LVOT(O), left ventricle outflow tract (obstruction); NHYA, New York Heart Association Classification, NPO, Nil per os; MAT, multifocal atrial tachycardia; MV, mitral valve; PRBC, packed red blood cells; PA, pulmonary artery; PAH, pulmonary arterial hypertension; PV, pulmonary Valve; PVS, pulmonary valve stenosis; RVOT(O), right ventricle outflow tract (obstruction); TPN, total parenteral nutrition.

In all reported cases, improvement of symptoms related to the primary indication was observed after trametinib initiation. Although the quality of the follow-up was heterogeneous and of variable duration, with a median duration of 13 months (4 m–24 m), we observed that among the 8/17 patients treated for a major lymphatic complication, 6/8 patients (Dori 2020, Hirbernik 2023, Nakano 2022 case 1 and 2, Leenders 2024, Hofbeck 2021) showed complete resolution, 2/8 patients presented a partial or transient improvement: our case (De Brouchoven 2024), whose treatment was discontinued because of an adverse event and patient #8 (Taliercio 2024) who responded only transiently to treatment. Among the 6/17 patients treated for concommitant lymphatic and cardiac manifestations, 4/6 patients (Lioncino 2022, Torok 2024, Meisner 2021, Andelfinger 2019 case 2) showed complete lymphatic resolution and a significant improvement in cardiac condition. Patient #9 (Pascarella 2024) had HCM improvement but PS persistence. Patient #11 (Nakano 2022 case 3) died after a transient improvement in lymphatic and cardiac symptoms (see discussion below). Finally, among the 3/17 patients presenting primarily with primary cardiac manifestations, patient #17 (Mussa 2021) died, patient #15 (Andelfinger 2019: case 1) showed a significant improvement and patient #16 (Leegaard 2022) showed complete resolution. A positive effect on growth was observed in 6/17 patients (Dori 2020, Nakano 2022 case 1-2, Andelfinger 2019 case 1-2, Leegaard 2022). The available data do not allow to accurately define the response time to treatment. Multifocal atrial tachycardia, which is frequently associated with NS and RASopathies, appear to respond rapidly, within 48–72 h (Lioncino 2022, Meisner 2021). Chylothorax and CPL appear to improve within the week following treatment (De Brouchoven 2024, Lioncino 2022), with a median resolution time of 3 months (0.75 m–6 m). The response time to treatment seems longer for HCM and CHD, with a median time to observe a significant response of 1.25 month (1 m–4 m), and complete resolution after a median delay of 17 months (14 m–21 m).

Three out of 17 patients died. Patient #8 (Taliercio 2024 case 2) showed an initial regression of chylothorax after trametinib was started but deteriorated again after 17 days and was redirected to palliative care. Patient #11 (Nakano 2022 case 3), in whom CPL, chylothorax and HCM had initially improved, died out of an unexplained acute cardiac event at home after 1 month of trametinib treatment. Patient #17 (Mussa 2022), born at 33 weeks of gestation who had severe hemodynamic dysfunction secondary to HCM and had been started on trametinib at 41 weeks corrected age, unexpectedly died out of respiratory deterioration after one month. The autopsy revealed capillary pulmonary hemangiomatosis as the cause of death. Non-critical adverse events were reported in 7/17 patients (De Brouchoven 2024; Hirbernik 2023; Nakano 2022, case2; Leenders 2024; Pascarella 2024; Meisner 2021; Leegaard 2022) including diarrhea, rectorrhagia and eczema. These events occurred with doses ≥0.025 mg/kg/day except for patient #6 (Leenders 2024), who presented mild eczema with a 0.018 mg/kg/day dosage. Adverse effects led to dosage reduction in 2/17 (Nakano 2022 case 2, Meisner 2021) and discontinuation in 1/17 (De Brouchoven 2024).

## Discussion

Current knowledge of MEK inhibitor pharmacology mostly derives from adult oncologic studies, including melanoma, thyroid cancer and non-small-cell lung cancer. Only a few formal clinical trials have been conducted in pediatric oncology, including trametinib plus dabrafenib (a *RAF* inhibitor) in glioma with *BRAF* V600 variants (42), and stand-alone trametinib in refractory juvenile myelomonocytic leukemia (43). Besides the NS cases reviewed here, trametinib has been used off-label on a case-by-case basis in Costello syndrome (44) and refractory epilepsy related to cardiofaciocutaneous syndrome (45).

### Efficacy

Trametinib appears to improve critical and/or refractory lymphatic and cardiac dysfunction associated with NS in neonates and infants, but patient data in this series are based on separate case reports with no controls. Although criteria for efficacy are often missing or not strictly defined in the reports, we considered a return to non-critical status (including extubation, chest tube removal or discharge from intensive care units) as a positive short-term outcome. However, sustained efficacy should be ascertained by assessing results on long term follow-up *RAF1* mutant mice modelling NS-associated HCM demonstrate a resolution of the cardiac and morphological phenotype with postnatal MEK inhibition, however, these mice study lack long-term follow-up for assessing the persistence of these effects with time (15). In a recent retrospective cohort study of 30 infants treated with trametinib for HCM, a sustained cardiac and clinical improvement was obtained and maintained after treatment weaning in 5 patients (46). Human studies are warranted to explore the long-term outcome of cardiac and pulmonary manifestations after an initial time-limited trametinib course, and the place of further courses in case of relapse. Animal studies support an efficacy of MEK inhibition in correcting non-critical NS features such as impaired growth and dysmorphism (15), and craniofacial and brain anomalies (25, 26). Given the relatively reassuring safety profile of trametinib in NS-affected infants and children, future studies should investigate the benefits and safety of early-onset treatment for the correction of noncritical NS features such as short stature, impaired neurodevelopment, endocrine, renal and skeletal anomalies that may nevertheless have a lifelong impact on quality of life.

The efficacy of trametinib does not appear to be gene-specific, as NS was caused by variants in four different genes in this series, but larger series are warranted in order to detect such differences. Lymphatic manifestations seem to respond quicker than cardiac ones. This may be related to the different natural course of the disease: primary chylous effusions typically have early onset, often at birth or during fetal life, then may regress, whereas HCM and valvular lesions often develop more gradually and persist later in infancy or childhood.

### Safety and adverse effects

Adverse events include skin disorders (the most frequently observed), vomiting, pancreatitis, hepatitis, colitis, ophthalmologic complications, cardiotoxicity, rhabdomyolysis, immune suppression, coagulopathy, thrombosis and others (47). In this series, whereas adverse effects were reported in 75% of the subjects who received doses ≥0.025 mg/kg, such effects were reported in only 11% of those with lower dosing; however, the available data do not allow to link the occurrence of adverse effects with treatment length. Skin side effects may be related to both dose and length of therapy. In a recent review of MEK inhibitors adverse effects in adult oncology patients, adverse skin effects often declare during the first month of treatment and persist with time in a dose-related manner (48). Adverse effects with trametinib in RASopathies appear to be less severe than in oncology patients. Several factors may contribute to these differences: higher trametinib dosing, frequent use of combination chemotherapies, but time-limited regimen in oncology patients (see below) (49, 50). It is important to mention that, for the treatment of RASopathy, MEK inhibition is not applied as a cytotoxic therapy with the aim to kill cells, but rather as a mean to bring RAS/MAPK signaling down to normal levels and restore regular cell functions (51). So, MEK inhibition for oncology therapy may down-regulate physiologic RAS/MAPK signaling to sub-normal level in healthy cells, which may account in part for the more severe adverse effects in these patients (20).

A close screening for these effects is advisable, as recommended by Nakano et al. who propose a monitoring protocol including bloodwork, eye exam, skin exam and cardiologic surveillance pre-treatment and for a duration of 48 weeks (31).

The death of 3 subjects in this series raises a concern for safety. Patient #8 (Taliercio 2024 case 2), a 29-week preterm infant with severe HCM and PS, presented at 3 months of age with a chylothorax that required an escalation of respiratory support and insertion of chest tube. As the infant failed to show a sustained improvement with trametinib, support was electively withdrawn in the context of other prematurity-related comorbidities. Thus, death is attributable to trametinib failure rather than an adverse pharmacological effect in this case. Patient #11 (Nakano 2022 case 3) had an apparently complete resolution of CPL but some degree of HCM persisted; thus, the sudden death after treatment discontinuation presumably derives from HCM-related arrythmia, and is less likely to be a direct side effect of trametinib. Patient #17 (Mussa 2021) died while on trametinib therapy, out of pulmonary capillary hemangiomatosis, a form of vascular lung disease typically associated with bi-allelic *EIF2AK4* variants in its heritable form (52) or secondary to various parenchymal lung disorders (53), but not typical associated NS. Parenchymal lung disease has been reported as a side effect of MEK inhibitors in adults (54), but whether this event was related to the drug itself, to chronic lung disease of prematurity or to a genetic cause is unknown.

### Dosage and regimen

As it is considered a rescue therapy, trametinib is often started with a significant delay, after standard management has failed. The median time from the onset of critical symptoms and the beginning of the treatment was 3.5 months, which may depend on various factors, such as delays in molecular diagnosis, in clinical decision-making and consenting parents, in obtaining appropriate approvals for off-label compassionate use, securing financial coverage, among other issues. As for patient #8 (Taliercio 2024 case 2), NS patients with early-onset HCM are at significant risk of acute cardiac events with a mortality rate as high as 22% at 1 year (55), underscoring the potential importance of early and sustained trametinib therapy.

Trametinib dosages ranged from 0.01 to 0.032 mm/kg/day, with gradual increase based on clinical response and tolerance. Data are lacking in order to affirm whether clinical response is dose-dependent. The dosage used in RASopathies is typically lower than in oncology, which can be explained by the fact that germline mutations in RASopathies often display a lower gain-of-function compared to somatic cancer mutations where higher ERK phosphorylation levels determine stronger hyperactivation of the cascade, thus require higher MEK inhibitor dosing (6, 7, 9, 37, 42, 51). Additionally, among NS patients, individual variants exhibit different gain-of-function levels, which result in various degrees of ERK phosphorylation. Finally, as NS is genetically heterogeneous, each mutated gene in the pathway may have a different impact on MEK activation, thus different drug responses (20, 27, 56). These differences likely explain why certain patients seem to respond to lower doses of MEK inhibitors, while others require higher doses to achieve therapeutic effects. This highlights the need for dose personalization based on the functional impact of specific variants.

Conversely, somatic mutations disappear with cancer cell death as a result of chemotherapy, while germline mutations will exert their effects for a lifetime, influencing the duration of treatment. Some patients in this series were treated for short durations with beneficial effects, such as patient #3 (Hirbernik 2023) who was treated for 3 months with satisfying results. However long-term follow-up data are lacking to estimate the type and recurrence of symptoms after treatment discontinuation. Given the off-label use, treatment should be discontinued once clinical improvement is deemed satisfactory or upon the occurrence of a major side effect. Nevertheless, prolonged use of trametinib has also shown beneficial effects on long-term growth. As an example, #16 (Leegaard 2022) was treated for 2 years with sustained growth improvement and no significant adverse effects. Based on the limited data available, we suggest that trametinib should be used as a second-line therapy for critical NS manifestations, as soon as possible after onset, and for a duration limited to a satisfactory control of these complications, typically from 3 months to 2 years (51, 55).

### Prevention of neonatal mortality and morbidity

Fetal anomalies are often present in RASopathies, and, if recognized, may allow for antenatal molecular testing. Nine to 16% of fetuses with increased nuchal translucency and a normal karyotype are identified with pathogenic NS variants, allowing very early diagnosis (57). Genetic testing in the presence of suggestive anomalies allow to identify RASopathies in up to 30% of affected fetuses as early as the second semester (14, 58, 59). Among these features, non-immune hydrops fetalis (NIHF) and pleural/pericardial effusions carry a perinatal mortality risk up to 30% (60). HCM, also a major risk factor for neonatal mortality and morbidity, can be detected from the 3rd trimester of fetal life in a subset of cases (61). Thus, precision diagnosis based on fast genetic testing, which is possible as early as the second trimester of pregnancy, offers a unique opportunity for fetal intervention, which may include targeted treatment with MEK inhibitors before critical/lethal complications such as chylothorax or hydrops occur.

Animal models of NS using *PTPN11* (26) and *KRAS* mutant mice (25) show that prenatal treatment with a MEK inhibitor rescues perinatal mortality and prevents cardiac and craniofacial defects. There are no adequate and well-controlled studies of trametinib in pregnant women, but animal studies have shown reproductive toxicity and teratogenicity, thus the European Medicines Agency (EMA) (https://www.ema.europa.eu) and the FDA (https://www.fda.gov) advise against trametinib use in pregnant women. In the few published case reports to date, no congenital malformations were described in newborns exposed to vemurafenib during pregnancy (62). Given the fetal and neonatal risks described above, pilot studies weighing benefits vs. safety of MEK inhibitor treatment in fetuses with critical NS complications should be considered.

### Limitations

The evidence presented in this review, limited by the nature and quality of patient data, mainly derived from single case reports (some of which only published as conference abstracts), should be considered as low according to standard evidence-based medicine guidelines (63). We sought to assess the effects of a specific treatment in a well-defined rare disease, conducted an exhaustive literature search, and applied a standard quality assessment method for inclusion. However, we cannot provide clinically applicable guidelines but only suggestions, as the small number of subjects, the lack of controls, and the variability of drug regimens limit the capacity to estimate positive and negative treatment effects. There is a high risk of selection bias, as subjects in whom the treatment was unsuccessful or the outcome unfavorable may be less likely to be published. Individual patient results appear consistent between studies, but the reporting is variable and, for some, imprecise (Table 1). Thus, this review should be only used to inform individual clinical decision making, and not as a formal guideline or recommendation.

## Conclusion

Based on the limited evidence available, this review suggests that trametinib is efficacious for the treatment of NS infants and children with severe/refractory respiratory or hemodynamic dysfunction, should be started early once the patient does not show a clear response to conventional management, should be started at a low dose (∼0.01 mg/kg/day) and increased stepwise to a target dose of 0.02–0.032 mg/kg/day with close monitoring for side effects, and should be discontinued once critical pulmonary and/or cardiac symptoms are controlled. Trametinib remains an experimental drug for this indication, and its availability is limited and subjected to manufacturer's approval and Single Patient Investigational Drug approval from regulatory bodies. Families should be properly informed of the expected benefits and risks, and consent for this off-label use of trametinib as rescue therapy.

If off-label use of targeted therapies has a solid molecular basis, it is faced with a limited clinical support, reimbursement challenges related to the very high pricing and the cost of genotyping (64). The high heterogeneity of genotypes and phenotypes, combined with the relative rarity of RASopathies, represent a challenge for systematic evaluation of treatments and a limit to clinical recommendations. Prospective clinical trials may be hampered by financial and logistic issues, as well as ethical considerations in a highly vulnerable population, as it is often the case with clinical research in rare diseases. Even if phase II randomized controlled trials are under way (for example: clinicaltrials.gov/study/NCT06555237), real-world data are increasingly recognized as a reliable source of evidence, if using appropriate study models and analysis methods, and overcoming issues in data heterogenicity, quality, and accessibility. In this regard, multicenter international registries represent fundamental building blocks for advancing research in MEK inhibition for RASopathies. Future research should explore the place of MEK inhibitors in preventing MS manifestations since fetal life and across the lifespan, and in improving the outcome of lifelong comorbidities.

## Data Availability

The original contributions presented in the study are included in the article/Supplementary Material, further inquiries can be directed to the corresponding author.

## Appendix 1: PRISMA 2020 flow diagram for reviews which included searches of databases

*Duplicates: Same articles in different databases or same patient reporting in different references.

**Selection criteria: age <18 years; trametinib as MEK inhibitor; genetic diagnosis of Noonan syndrome; primary cardiac or lymphatic presentation.

***References:

1. Fabri L, Tobia A, Ludemann J, Rathgeber S, Kieran E, Nguyen T, et al. Trametinib and Lymphangiectasia in Noonan Syndrome: A Success Story. A62 PULMONARY INVOLVEMENT IN PEDIATRIC SYSTEMIC DISEASE: American Thoracic Society; 2024. p. A2282-A.

2. Wiedl C, Bornhorst M, Cheng J, Jacobsohn D A case report of myelodysplastic syndrome in a patient with PTPN11-related Noonan syndrome. Pediatric Blood & Cancer. 2024;71(7):e30948.
